# Continuous Epidural Analgesia (CEA) via Single Catheter Providing Profound Analgesia for Pediatric Patients Following Posterior Spinal Fusion (PSF) in Adolescent Idiopathic Scoliosis (AIS)

**DOI:** 10.7759/cureus.37066

**Published:** 2023-04-03

**Authors:** Lloyd M Halpern, Abby R Velarde, De-An Zhang, William Bronson, Clark Kogan

**Affiliations:** 1 Pediatric Anesthesia, Shriners Children's Spokane, Spokane, USA; 2 Medicine, Washington State University, Spokane, USA; 3 Pediatric Anesthesia, Shriners Children's Southern California, Pasadena, USA; 4 Orthopaedics, Shriners Children's Spokane, Spokane, USA; 5 Statistics, Washington State University, Spokane, USA

**Keywords:** post-operative opioids, posterior spinal fusion and instrumentation, continuous epidural infusion, pain management, adolescent idiopathic scoliosis

## Abstract

Introduction: Posterior spinal fusion (PSF) is a commonly performed orthopedic procedure to correct scoliosis in children. Continuous epidural analgesia (CEA) is a proposed means of providing analgesia following PSF. Whether a single epidural catheter with the tip in the upper thorax can provide adequate analgesia for PSF, which often spans the upper thoracic to lower lumbar regions, is unresolved in the literature.

Method: In this single-center, retrospective study, we reviewed 69 consecutive patients undergoing PSF for adolescent idiopathic scoliosis (AIS) with CEA at our institution from October 1, 2020 to May 26, 2022. Data for the entire cohort was divided into two time intervals before and after epidural removal, group epidural (Epi) and group no epidural (No Epi). Daily intravenous and oral opioid morphine equivalents per kilogram (OME/kg) plus mean and maximal visual analogue pain scores (VAS 0-10) were recorded from post-anesthesia care unit (PACU) discharge to the end of postoperative day (POD) three.

Results: 57 patients were included in the study. Opioid usage was 4.5 times greater in the 19 hours following removal of the epidural catheter when compared to the entire period (mean 65 hours) the epidural was in place (Group Epi 0.154 OME/kg vs Group No Epi 0.690 OME/kg, p<0.001). 51% (29/57) of patients did not require opioids (intravenous or oral) while the epidural was in place, all patients required opioids after epidural removal. Mean opioid usage while the epidural was in place was 9.3 OME, equivalent to approximately 6 mg of oxycodone. Mean and maximum pain scores increased significantly after removal of the epidural on POD 3 (mean pain score: Epi 3.4 (1.8) vs No Epi 4.1 (1.7); p<0.001) (max pain score: Epi 4.9 (2.5) vs No Epi 6.3 (2.1); p<0.001).

Conclusions: This is the first study we are aware of to report pain scores and cumulative opioid requirements for PSF patients receiving CEA with a single epidural catheter before and after epidural removal. Opioid usage increased over four times in the 19 hours after epidural removal compared to the total opioid requirements while the epidural was infusing. Mean and maximum pain scores increased significantly after removal of the epidural on POD 3. This study firmly establishes that CEA with a single epidural catheter can provide profound analgesia for patients having PSF for AIS.

## Introduction

Posterior spinal fusion (PSF) is a commonly performed orthopedic procedure to correct scoliosis in children. It is associated with substantial pain and opioid use in the postoperative period. Continuous epidural analgesia (CEA) with local anesthetic delivered via a single epidural catheter was first proposed as a means of providing analgesia following PSF in 1996 [1]. Whether a single epidural catheter with the tip in the upper thorax can provide adequate analgesia for PSF, which often spans the upper thoracic to lower lumbar regions, is unresolved in the literature. Studies examining spread of contrast medium in the thoracic epidural space (injection level T3-T5) report a maximal spread of 11 vertebral segments (mean 5.1 segments, T3-T8) following the injection of 8 ml of iohexol (OMNIPAQUE) [2]. Multiple studies have provided conflicting data on the analgesia provided by CEA following PSF. Five previous studies found no difference in pain scores when comparing CEA using a single epidural catheter with patient-controlled analgesia (PCA) for PSF [3-6]. Two studies reported a significant improvement in pain scores when comparing a single catheter CEA with PCA [7,8]. However, patients in these studies had free access to opioids in addition to the CEA and opioid usage was not reported. A subsequent meta-analysis reported that pain scores were lower in the epidural group only when two epidural catheters were used, one at the superior and inferior border of the incision [9,10]. A study comparing one and two epidural catheters to PCA found that pain was significantly reduced only with two epidural catheters [11]. The purpose of this study is to compare opioid requirements and pain scores before and after removal of a single epidural catheter to assess the level of analgesia provided by CEA delivering a local anesthetic/opioid mixture. We hypothesized that opioid usage and pain scores would significantly increase after removal of the epidural catheter.

## Materials and methods

In this single-center, observational, retrospective study, we reviewed 69 consecutive patients undergoing PSF for adolescent idiopathic scoliosis (AIS) at our institution from October 1, 2020 to May 26, 2022. Approval was obtained for all parts of the study by the Washington Institutional Review Board, Olympia, USA. Patients who had PSF for diagnoses other than AIS, underwent previous spine surgery, or did not have an epidural for postoperative analgesia were excluded from the study. We collected demographic data for all participants to include age, gender, weight, Cobb angle, surgical duration, and number of levels fused. Daily intravenous and oral opioid requirements plus mean and maximal visual analogue pain scores (VAS 0-10) were recorded from post anesthesia care unit (PACU) discharge to the end of postoperative day (POD) three. Opioid usage was converted into oral morphine equivalents per kilogram (OME/kg) because of the wide range of weights in our study population. Pain score measurements were taken a minimum of every four hours. The mean pain score for the day was determined by summing the pain scores and dividing by the number of observations. Pain scores recorded as zero during sleep were not included . Data for the entire cohort was separated into two time intervals. The first interval began after discharge from the PACU and ended with removal of the epidural catheter at 0500 on POD 3 (Group Epi). The second time interval began after removal of the epidural catheter and ended at midnight on POD 3 (Group No Epi). The primary outcome is the comparison of opioid usage while the epidural was in place and after epidural removal. Secondary outcomes include the number of patients not requiring opioids with the epidural in place and after epidural removal, pain scores in the two group, the number of patients reporting severe pain (VAS >7) all three PODs, and complications associated with the epidural catheter.

All epidural catheters were placed intra-operatively by the surgeon with the tip of the catheter measured to reach the fifth thoracic vertebral level. The epidural space was identified under direct vision between vertebral levels T8 to L1. A 16 gauge, 5.25 inch angiocatheter was advanced superiorly into the epidural space through a small midline laminotomy and the epidural catheter threaded through the angiocatheter. The angiocatheter was then removed and the distal end of the catheter was tunneled through the paraspinal muscles and out through the skin lateral to the incision. An epidural test dose was performed prior to wound closure. Following confirmation of spontaneous movement of both lower extremities, the epidural was dosed with a bolus of local anesthetic determined by the attending anesthesiologist. All epidural infusions contained 0.1% bupivacaine with 5 mcg/mL hydromorphone or 0.1 % ropivicaine with 5 mcg/mL of fentanyl. The infusion was initiated upon arrival in the PACU. The rate of the epidural infusion was at the discretion of the attending anesthesiologist. The rate of infusion and any changes to the infusion rate were collected. Once satisfactory analgesia was achieved the patient was transferred to the inpatient unit.

No changes in our analgesic protocol occurred during the study period. Patients received a multi-modal analgesia regimen with scheduled acetaminophen (15 mg/kg oral/intravenous q6 hours), a non-steroidal anti-inflammatory drug (ketorolac 0.5 mg/kg intravenous or ibuprofen 10 mg/kg oral q6 hours), and gabapentin (5 mg/kg oral q8 hours, no loading dose). Diazepam (0.1 mg/kg intravenous q6 hours) or methocarbamol (750 mg intravenous q6 hours) were available as needed for the treatment of muscle spasm. Oxycodone (0.1 mg/kg oral q4 hours) and morphine (0.1 mg/kg intravenous q hours) were used as needed after discharge from the PACU for breakthrough pain of four or greater. Patients who could not tolerate morphine or oxycodone were given intravenous or oral hydromorphone. All patients received similar postoperative standardized physical therapy and rehabilitation measures. All patients had their epidural infusions turned off at 0500 on POD 3 and routinely went home on POD 4.

Midway through the study period, we transitioned from a flexible, radiolucent, nylon epidural catheter (Perifix, B. Braun, Bethlehem, USA) to a radiopaque, wire-reinforced, styletted Arrow TheraCath epidural catheter (Teleflex, Morrisville, USA). Subsequently, the vertebral level and the distance from the midline of the Theracath epidural tip was confirmed on the postoperative chest x-ray. We compared opioid usage and pain scores with both types of epidural catheters.

All patients received a balanced general anesthetic with propofol and remifentanil infusions plus 2% desflurane. Fentanyl, morphine, and hydromorphone were used for analgesia intraoperatively and in the PACU as determined necessary by the attending anesthesiologist per their usual practice. Neuromonitoring was employed in all cases. Foley catheters were inserted intraoperatively and were discontinued at the time of epidural removal.

Statistical analysis

Demographics, pain scores and opioid requirements distributions are summarized with mean and standard deviation. Unpaired t-tests were used to compare demographics, pain scores and opioid requirements among epidural catheters. Paired t-tests were used to compare pain scores and opioid requirements across time. The chi-square test was used to compare the incidence of use of opioids (yes/no) between groups.

## Results

Of the 69 patients reviewed, 12 were removed for meeting one of the exclusion criteria (eight neuromuscular patients, three MAGnetic expansion control (Magec) growing rods, one anterior spinal fusion). Patient demographics and surgical parameters of the remaining 57 patients are presented in Table 1. 

**Table 1:** Demographic data and surgical parameters

Variable	Entire Cohort	Perifix (P)	Theracath (T)	P value (P vs T)
Age (years) (SD)	14.8 (2.3)	15.0 (1.8)	14.8 (2.6)	0.38
Gender M/F (% female)	13/44 (77.2%)	7/23 (76.7%)	6/21 (77.8%)	0.92
Weight (kg) (SD)	60.7 (17.6)	62.5 (6.5)	59.6 (18.9)	0.55
Cobb angle (degrees) (SD)	55.8 (8.7)	55.1 (8.3)	57.2 (9.6)	0.41
Levels fused (SD)	9 (1.5)	9 (1.4)	9.2 (1.5)	0.62
Surgical duration (hours:minutes) (SD)	3:04 (45)	3:10 (41)	3:00 (47)	0.39
Epidural catheter type	57	30	27	N/A
Epidural infusion rate (mL/hr) (SD)	6.1 (1.2)	6.7(1.6)	5.9 (1.4)	0.36
Distance epidural tip from midline (mm) (range)	N/A	N/A	0.6 (0-120)	N/A
Distance epidural tip from T5 vertebrae (mm)	N/A	N/A	0.7	N/A

For the primary outcome, opioid usage was 4.5 times greater in the 19 hours following removal of the epidural catheter when compared to the entire period (mean 65 hours) the epidural was in place (Group Epi 0.152 OME/kg vs Group No Epi 0.684 OME/kg, p<0.001). Table 2 presents total oral and parenteral daily opioid usage. 

**Table 2:** . Opioid usage by day in the epidural and non-epidural groups DOS: Day of surgery from after PACU discharge until midnight; POD: Post-operative day *p < 0.05

	Epidural				No Epidural
	DOS	POD 1	POD 2	POD 3 before 5 am	POD 3 after 5 am
Number of patients using opioids (%)	7/57 (12%)	7/57 (12%)	22 (38%)*	12 (21%)	57 (100%)*
Number using opioids compared to previous day p value	n/a	1	0.004	0.09	<0.001
Total opioids OME/kg	0.007	0.029*	0.087*	0.030	0.690*
OME/kg compared to previous day p value	n/a	0.03	<0.001	0.10	<0.001 vs POD 2

In the entire cohort, 51% (29/57) of patients did not require opioids (intravenous or oral) while the epidural was in place. The mean total opioid usage while the epidural was in place was 9.3 OME, equivalent to approximately 6 mg of oxycodone for the 65 hours the epidural was infusing. Two patients required a total of 4 mg of intravenous morphine while the epidural was in place. The remainder of the opioids were oral while the epidural was in place. 88% of patients (50/57) did not require any opioids from after discharge from PACU to POD 1. 38% (22/57) of patients used opioids on POD 2. All patients (57/57) required opioids after epidural removal on POD 3. Two patients required intravenous morphine after epidural removal, the remainder were oral. Opioid usage showed evidence of an increasing trend across each POD. The largest increase in opioid use while the epidural was in place was between the DOS and POD 1. Patients received a mean of 870 mcg of opioid (hydromorphone or fentanyl) via the epidural for the duration the epidural was in place (mean rate epidural infusion 6 mL/hr).

Mean and maximum pain scores increased after removal of the epidural on POD 3 (mean pain score: Epi 3.7 (1.6) vs No Epi 4.1 (1.6); p=0.005) (max pain score: Epi 5.3 (2) vs No Epi 6.2 (2.1); p<0.001). Table 3 reports daily pain scores. 

**Table 3:** Pain scores by day in the epidural and non-epidural groups DOS: Day of surgery from PACU discharge until midnight; POD: Post-operative day *p < 0.05

	Epidural				Non-Epidural
Pain Scores	DOS	POD 1	POD 2	POD 3 < 5 am	POD 3 > 5 am
Mean (SD)	4.0 (2.1)	3.2 (1.8)*	3.4 (1.8)	4.1 (1.7)*	4.1 (1.6)*
Mean pain compared to previous day p value	n/a	<0.001	0.11	<0.001	<0.001
Max (SD)	5.6 (2.7)	4.7 (2.3)*	4.9 (2.5)	6.1 (2.1)*	6.3 (2.1)*
Max pain compared to previous day p value	n/a	0.002	0.18	<0.001	<0.001 vs POD 2

Maximum pain scores were greatest on POD 3 after epidural removal. The mean pain score on POD 3 after epidural removal was significantly higher than POD 1 and POD 2, but did not reach statistical significance when compared to the mean pain score on the DOS. Pain scores were similar on POD 1 and POD 2, although opioid usage showed an increase on POD 2 (p=0.03).

We found no evidence that the two catheter groups were unbalanced with respect to age, gender, magnitude of the curve, surgical duration or number of levels fused. We found no differences in pain scores and opioid usage when comparing the flexible (Perifix) and stiff, radiopaque (Theracath) epidural catheters (Table 4). 

**Table 4:** Opioid usage and pain scores in the Perifix and Theracath epidural catheter groups

Catheter type	Perifix	Theracath	P value
Number not requiring opioids (%)	12/30 (40%)	17/27 (63%)	p=0.08
Opioid usage (OME/kg)	0.18 (0.22)	0.12 (0.27)	p=0.41
Average pain score (SD)	3.6 (1.9)	3.6 (1.6)	p=0.45

We did find suggestive evidence of a decrease in the incidence of breakthrough pain in the Theracath group with 63% (17/27) not requiring opioids while the epidural was in place compared to 40% (12/30) in the Perifix group (p = 0.08). 12% patients (7/57) reported at least one episode of severe pain (VAS >7) while the epidural was in place each of the three PODs, four in the Perifix group and three in the Theracath group. The three in the Theracath group reporting severe pain each POD had epidural catheters with the tip within one vertebral segment of the fifth thoracic vertebrae and within 6 mm of the midline. The mean distance of the Theracath epidural from the midline was 0.6 mm (range 0-12 mm). The mean distance of the catheter tip from T5 was 0.7 vertebral segments (range 0-3).

67% patients (38/57) had a side effect that required treatment while the epidural was infusing (Table 5). 

**Table 5:** Complications requiring treatment associated with CEA and new complications after epidural removal CEA: Continuous epidural analgesia

Side Effect	Number	Percentage
Nausea and vomiting	29/57	50.9%
Pruritis	9/57	15.8%
Respiratory depression	1/57	1.8%
Sedation	4/57	7.0%
Hypotension	3/57	5.2%
Horner’s syndrome	1/57	1.8%
Neurologic symptoms	2/57	3.5%
After removal:		
Nausea and vomiting	11/57	19.3%
Muscle spasm	5/57	8.8%
Pruritis	1/57	1.8%

The most common adverse events were nausea or vomiting in 51% and pruritus in 16% of patients. One patient developed Horner’s syndrome and two patients developed numbness (one of an arm, one in both legs) that resolved after the infusion was stopped. All three of these infusions were subsequently restarted. Seven patients required a rate reduction for excessive sedation or mild hypotension. There were no episodes of hypotension that required pharmacologic treatment. 14 patients required a rate increase for inadequate analgesia. 30% patients (17/57) developed a new side effect not present before epidural removal when an opioid was first initiated (Table 5). No epidurals were discontinued prematurely. There were no persistent neurologic complications in any patient.

## Discussion

This is the first study we are aware of to report cumulative opioid requirements for PSF patients using CEA with a local anesthetic/opioid mixture via a single epidural catheter and following its removal. The results of previous studies comparing single and double epidural catheter CEA with opioid-based analgesia for postoperative pain control after PSF are presented in Table 6.

**Table 6:** Results previous studies comparing CEA and opioid-based analgesia for PSF CEA: Continuous epidural analgesia; PSF: Posterior spinal fusion

Author	Year	Number catheters	Infusate	Number patients	Pain scores with epidural	Opioid usage with epidural
Cassady et al. [4]	2000	One	Local	33	No difference	N/A
Van Boerum, Smith, Curtin [3]	2000	One	Local	50	No difference	N/A
O'Hara et al. [5]	2004	One	Local	31	No difference	No difference
Blumenthal et al. [10]	2005	Two	Local	30	Reduced	Reduced
Sucato et al. [7]	2005	One	Local	613	Reduced	N/A
Gauger et al. [6]	2009	One	Local	38	No difference	No difference
Milbrandt et al. [8]	2009	One	Local	138	Reduced	N/A
Klatt et al. [11]	2013	One and Two	Local	66	Reduced with 2 only	Reduced only with 2
Hong et al. [12]	2016	One	Hydromorphone	40	Reduced	N/A

More recent studies evaluating CEA for PSF have focused on the use of only opioids via the epidural [12,13]. All three previous studies evaluating CEA with a local anesthetic mixture via two simultaneous epidural catheters have shown a reduction in pain scores compared to OBA [9-11]. However, patients with two epidural catheters cannot ambulate with the catheters in place because of lower extremity weakness associated with the lumbar catheter. The evidence for a single thoracic epidural catheter providing adequate analgesia with a local anesthetic mixture has been inconclusive. Data from studies examining the spread of radiopaque dye in the thoracic epidural space suggest that there is insufficient spread of local anesthetic to cover the length of the surgical procedure. The primary finding of this study is that CEA with a local anesthetic mixture via a single epidural catheter for PSF can provide profound analgesia. Discontinuing CEA on POD 3 is associated with a dramatic increase in opioid use and pain scores. Nearly two of three patients in the Theracath epidural catheter cohort, and one-half of the entire epidural cohort, did not use opioids during the time the epidural was infusing after discharge from PACU. The mean opioid usage in the entire cohort was equivalent to approximately 6 mg of oxycodone for the 65 hours the epidural was infusing. Opioid usage increased over four times in the 19 hours after epidural removal compared to the total opioid requirements while the epidural was infusing.

When assessing the analgesic effect of regional anesthesia on acute post-operative pain, it is important to consider both pain scores and opioid usage. In this study, we found no significant change in pain scores between POD 1 and POD 2. However, there was evidence of an increase in opioids on POD 2. Had we only evaluated pain scores we would have not observed this increased pain intensity on POD 2.

Although the epidural provided impressive analgesia, 12% (7/57) of the entire cohort experienced at least one episode of severe pain (>7) all three PODs the epidural was in place. Sucato et al. reported 13% of their epidural catheter cohort had their epidural prematurely removed, inadequate analgesia the reason in 61% of cases that had to be terminated [7]. They speculated that incorrect epidural position was the reason for epidural failure. We found that epidural position had no effect on pain scores or opioid usage in the Theracath group, but all catheters were within three vertebral bodies of the fifth thoracic vertebrae. These patients could represent a high pain-reporting cohort regardless of analgesic method or some yet undefined issue with epidural effectiveness.

We found that a wire-reinforced, styletted epidural catheter can reliably be placed at or near a specific vertebral level in the thoracic epidural space when placed under direct vision via an angiocatheter. Using a radiopaque epidural catheter allows identification of the catheter location and in cases where advancing the catheter is difficult, steering the catheter into position is possible. Further, we demonstrated that the Theracath catheter consistently does not deviate far from the midline in scoliotic patients. The Theracath epidural catheter has a spring wire tip to discourage intravascular and subarachnoid placement. There is a case report of a knot in the spring wire tip which occurred in 1988 [14]. This complication has not been reported since. We found an increased number of patients did not require opioids in the Theracath group, although this did not reach statistical significance. While the cumulative opioid usage did not differ, the Theracath group was less likely to require opioids for breakthrough pain. We speculate that this may be due to the Theracath catheter reaching the desired T5 level more consistently, although we were unable to determine the location of the radiolucent Perifix catheter to compare catheter position.

The use of CEA was associated with a significant number of side-effects requiring treatment. These results are consistent with a previous report [7]. All side-effects were manageable and none required discontinuation of CEA. Our single episode (2%) of respiratory depression requiring naloxone is lower than previously reported (7.3%), likely due to our reduced concentration of opioid in the epidural infusion (5 mcg/mL) compared to earlier studies (up to 20 mcg/mL) [7]. The rare occurrence of numbness did not adversely affect any patient outcome and all symptoms resolved quickly with discontinuing the infusion. The use of a foley catheter during the epidural infusion is standard. Patients do not complain about the urinary catheter because of the analgesia provided by the epidural infusion. We found no evidence of urinary tract infections.

This study has limitations inherent in an observational, retrospective study. It was performed in a single-center and requires a careful comparison with multiple centers. We lacked a control group that did not have an epidural infusion, making it impossible to make any conclusions on the effect of CEA compared to someone who did not receive an epidural. However, in comparing opioid usage and pain scores before and after epidural removal each patient served as their own control. There may have been other confounders but the observed differences in pain scores and opioid usage after epidural removal were highly significant.

## Conclusions

In conclusion, this study firmly establishes that CEA with a local anesthetic mixture via a single epidural catheter can provide profound analgesia in patients having PSF for AIS with low risk. We further demonstrate that a wire-reinforced, styletted epidural catheter can reliably be placed intraoperatively under direct vision to the desired position in the thoracic epidural space with radiologic confirmation of position. Future work will focus on studying the efficacy of CEA compared to intrathecal or parenteral only opioid-based protocols.
